# G-quadruplexes may determine the landscape of recombination in HSV-1

**DOI:** 10.1186/s12864-019-5731-0

**Published:** 2019-05-16

**Authors:** Nandhini Saranathan, Banhi Biswas, Anupam Patra, Perumal Vivekanandan

**Affiliations:** 1 Kusuma School of Biological Sciences, Indian Institute of Technology Delhi, New Delhi, India; 2 International Centre for Genetic Engineering and Biotechnology, New Delhi, India

**Keywords:** Herpes simplex virus-1, G-quadruplexes, Recombination breakpoints, Higher-order G4 s, G4- clusters

## Abstract

**Background:**

Several lines of evidence suggest that recombination plays a central role in replication and evolution of herpes simplex virus-1 (HSV-1). G-quadruplex (G4)-motifs have been linked to recombination events in human and microbial genomes, but their role in recombination has not been studied in DNA viruses.

**Results:**

The availability of near full-length sequences from 40 HSV-1 recombinant strains with exact position of the recombination breakpoints provided us with a unique opportunity to investigate the role of G4-motifs in recombination among herpes viruses. We mapped the G4-motifs in the parental and all the 40 recombinant strains. Interestingly, the genome-wide distribution of breakpoints closely mirrors the G4 densities in the HSV-1 genome; regions of the genome with higher G4 densities had higher number of recombination breakpoints. Biophysical characterization of oligonucleotides from a subset of predicted G4-motifs confirmed the formation of G-quadruplex structures. Our analysis also reveals that G4-motifs are enriched in regions flanking the recombination breakpoints. Interestingly, about 11% of breakpoints lie within a G4-motif, making these DNA secondary structures hotspots for recombination in the HSV-1 genome. Breakpoints within G4-motifs predominantly lie within G4-clusters rather than individual G4-motifs. Of note, we identified the terminal guanosine of G4-clusters at the boundaries of the U_L_ (unique long) region on either side of the OriL (origin of replication within U_L_) represented the commonest breakpoint among the HSV-1 recombinants.

**Conclusion:**

Our findings suggest a correlation between the HSV-1 recombination landscape and the distribution of G4-motifs and G4-clusters, with possible implications for the evolution of DNA viruses.

**Electronic supplementary material:**

The online version of this article (10.1186/s12864-019-5731-0) contains supplementary material, which is available to authorized users.

## Background

Herpes simplex virus-I (HSV-1), also known as Human Herpes Virus-I (HHV-1), is a double-stranded DNA virus with a genome size of about 150 kb. HSV-1 infects the epithelial (skin, mucosa) and neuronal tissues. The genome of HSV-1 is organized as long (L) and short(S) segments. Each segment (i.e. the L and the S segment) further comprises a central unique region (U) flanked by repeats (terminal repeats –TR; inverted repeats –IR) inverted with respect to each other. The HSV-1 genome is canonically represented as: TR_L_-U_L_-IR_L_-IR_S_-U_S_-TR_S_. This genomic arrangement allows unique regions to invert, thus resulting in four isomeric forms of HSV-1 genome. It is widely reported that the junction between IRL and IRS, known as the ‘a’ region, promotes the intramolecular recombination leading to isomeric genomes [1–3]. Intermolecular recombination in HSV-1 is, however, less well-studied.

G-quadruplexes (G4 s) are nucleic acid secondary structures formed by a sequence motif consisting of four guanine trinucleotides interspersed by nucleotides of atmost 15 bases in length. They exert spatio-temporal effects on transcription, translation, replication, telomere extension and alternative splicing in the mammalian and microbial genomes [4–6].

Many are the evidences for potential involvement of G4sin recombination. Recombination hotspots in human genome, loci of antigenic variation in microbial genomes, nucleotide segments associated with fragility and chromosomal translocations in cancer, are all known to be spatially associated with G4 s [7–10]. The proximity of G4 s to such sites of recombination may be relevant in the recruitment of protein factors necessary for the genetic rearrangement [11–13]. Among viruses, a role for G4 s in recombination has been studied only in HIV-1. Dimerization of HIV-1 genomes by formation of intermolecular G4 s in U3 region, DIS (dimerization site) and cPPT (central polypurine tracts) has been linked to switching-over of templates by reverse transcriptase in vitro, suggesting a role for G4 s in HIV-1 recombination [14–16].

The association of G4 s with recombination in the human genome is well-studied [8, 17, 18]. Although recombination is well-documented among DNA viruses infecting humans, the role of G4 s, if any, in the recombination of DNA viruses has not been investigated. We chose to investigate the role of G4 s in recombination in HSV-1 as (a) Several G4 s have been reported in HSV-1 [19–21] and (b) recombination among DNA viruses is most extensively studied in HSV-1 [22]. In addition, Lee etal (2015) recently studied the recombination of two HSV-1 strains, OD4 and CJ994, both in in vitro and in in vivo conditions; they characterized the exact nucleotide position of 577 breakpoints by sequencing of 40 recombinant HSV-1 strains (Additional file 1: Table S1) and mapped them to HSV-1 strain 17 [23]. The availability of 40 whole genome sequences of HSV-1 with over 500 intermolecular recombination breakpoints provided us with a unique opportunity to study whether a spatial association between recombination breakpoints and G4 s exists in the HSV-1 genome.

## Results and discussion

### The distribution of recombination breakpoints mirrors G4 densities in the HSV-1 genome

**Fig. 1:**
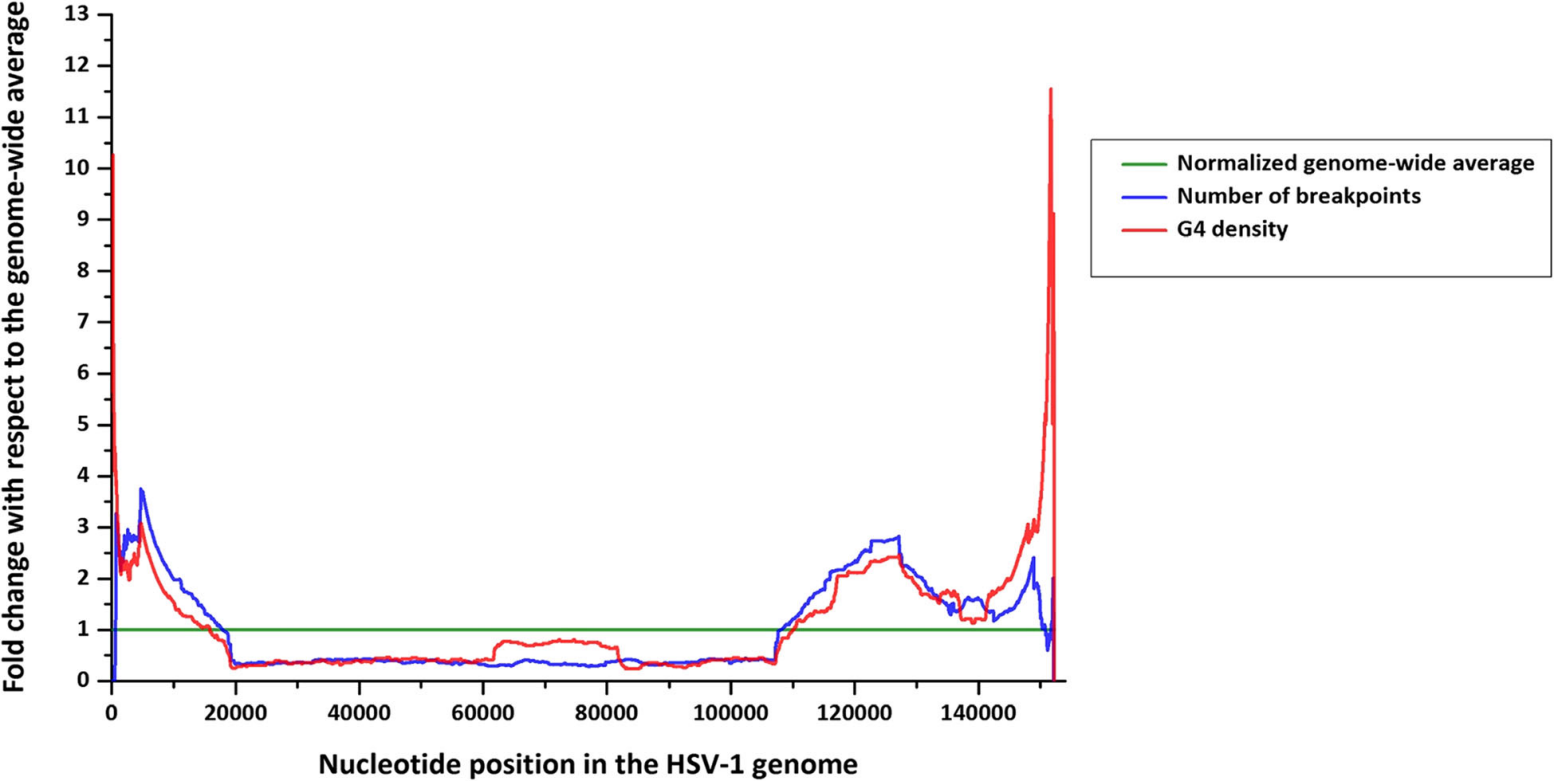
Genome-wide distribution of recombination breakpoints mirrors G4 densities in HSV-1. A nucleotide window of 100 bp was slid across the genome of HSV-1 strain 17 (Genbank accession number: NC_001806) at a step size of 1 bp (i.e a total of 152162 windows were analyzed for a genome that is 152,163 bp long). The G4 density and the number of breakpoints in each window were computed; the fold difference over their respective genomic averages was calculated window-wise and smoothened line graphs are plotted. The green line at 1 represents the normalized genome-wide average and demarcates the regions of local enrichment (above the green line) and depletion (below the green line). The blue and red lines superimpose each other genome-wide, indicating a spatial association between recombination breakpoints and G4-motifs in the HSV-1 genome

First, we sought to analyze the co-distribution of G4-motifs and recombination breakpoints, if any, by a sliding window method. Using an in-house program [19] (please see Methods section and Fig. 1 legend for description), a 100 bp window was slid along the length of the genome of HSV-1 strain 17. Quadparser was used for identification of G4-motifs and computation of G4 density window-wise. The red line in Fig. 1 represents the fold change in the G4 density of each window as compared to the overall G4 density (i.e. the average G4 density across all the windows). The blue line in Fig. 1 is the plot of the normalized number of breakpoints in each window (i.e the fold difference in the number of breakpoints in a given window as compared to the overall average across all windows). Upon overlay of the two line graphs, we observed clustering of recombination breakpoints over regions of high G4 density. In other words, regions containing more breakpoints had higher G4 density (Fig. 1). Similarly, recombination breakpoints are infrequent in regions of genome that are sparsely populated with G4-motifs. This spatial overlap of breakpoints and G4-motifs lends us to hypothesize that G4 s have functional roles in recombination in HSV-1.

### Enrichment of G4-motifs in regions flanking recombination breakpoints

**Fig. 2:**
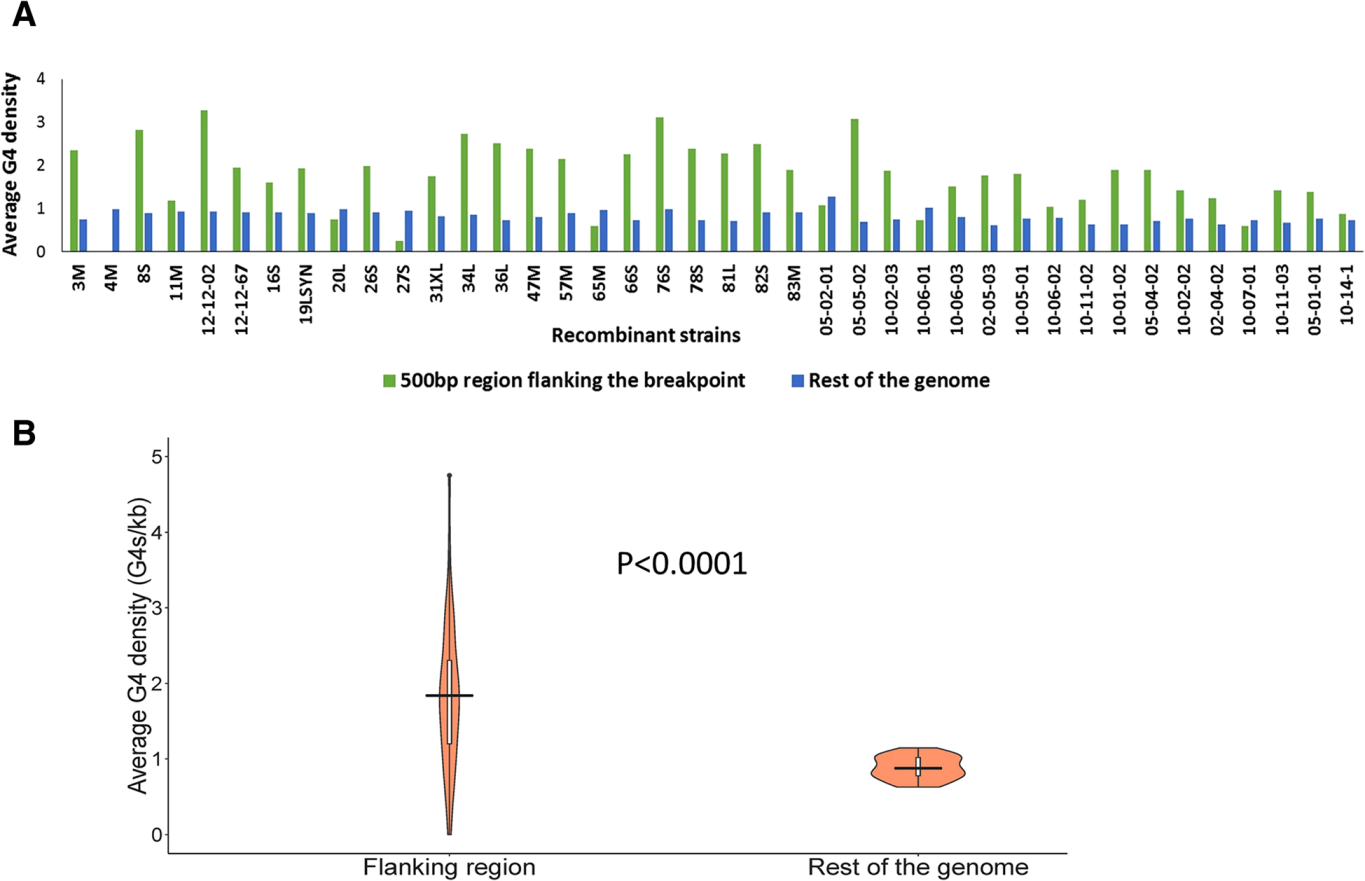
Flanking sites (500 bp on both sides) of recombination breakpoints are enriched for G4-motifs. **a** In majority of the recombinant strains analyzed, the average G4 density in the 1-kb bins centered on the recombination breakpoints is higher than that in the rest of the genome, indicating specific enrichment of G4-motifs near recombination breakpoints. **b** The violin plot summarizes the data from all 40 strains

As a more refined approach, the number of G4-motifs in the 500 bp region flanking (on both sides) each of the recombination breakpoints was obtained strain-wise using Quadparser [24]. The choice of 500 bp length (on both sides of recombination breakpoints) is based on the previous studies investigating the role of non-B-DNA structures in recombination events in the human genome and other eukaryotic genomes [9, 25, 26]. Among HSV-1 recombinant strains analyzed here, the average G4 density of regions flanking the breakpoints was found to be significantly higher than that of the rest of the genome (Fig. 2; *P* < 0.0001), indicating selective enrichment of G4-motifs near recombination breakpoints in HSV-1.

Given that most breakpoints lie in repeat regions, it is possible that inherent differences in nucleotide composition of genomic regions within the HSV-1 genome may influence G4 densities. To investigate this possibility, we randomized the sequences of the flanking regions for each breakpoint 5 times in all the 40 HSV-1 recombinants (i.e. a total of 577 breakpoints) studied; this was done without changing the overall nucleotide composition of the randomized sequences. The median G4 density of the native flanking regions was found to be significantly higher than the median G4 density of the randomized sequences (Additional file 1: Figure S1; *P* < 0.0001), indicating the selective enrichment of G4-motifs near recombination breakpoints is independent of differences in mononucleotide composition of the flanking regions. Taken together, we infer that the breakpoints in the HSV-1 genome are localized in the vicinity of G4-rich genomic segments. G4-motifs within 500 bp of breakpoints have been suggested to be functionally relevant in the chromosomal rearrangement in cancer [9, 25]. Furthermore, G4 s are enriched within a 500 bp flanking region of double-stranded breaks (DSBs) in *Saccharomyces cerevisiae* [26]. Our findings that G4-motifs are enriched in 500 bp flanking regions of recombination breakpoints corroborate the spatial relationship between G4 s and site of recombination in HSV-1 genomes.

It is well established that recombination and replication go hand-in-hand in HSV-1 [1, 27, 28]. Artusi et al reported the formation of G4 s in HSV-1 genome in concert with the virus’ replication cycle [29]. Taken together, a conjoint theory of a ternary temporal association among replication, recombination and G4 s can be conceived and the work presented here supports this notion.. ICP8 and UL12 constitute a two-component recombinase system in HSV-1 [30]. Of these two components, ICP8 is known to co-localize with G4 s during HSV-1 replication [29]. The HSV-1 encoded UL12 binds the tripartite MRN complex that is capable of binding G4 s [13, 31] . Several host encoded recombination and repair proteins are reported to be essential for HSV-1 recombination [32]. In light of these reports, the enrichment of G4-motifs in the flanking regions of breakpoints hints on their possible involvement as scaffolds that recruit the viral and host factors comprising the molecular machinery of recombination in HSV-1. The possible association between G4-motifs and recombination among other human herpes viruses (HHVs) merits further research.

### Biophysical characterization of G4-motifs flanking the recombination breakpoints

To verify whether the G4-motifs predicted by Quadparser to lie within the 500 bp region flanking the breakpoints truly formed the G4 structure, eight G4-motifs from the recombinant genomes were chosen randomly (Additional file 1: Table S2 and Table S3) for in vitro biophysical characterization by circular dichroism (CD), and nuclear magnetic resonance spectroscopies (NMR).

**Fig. 3:**
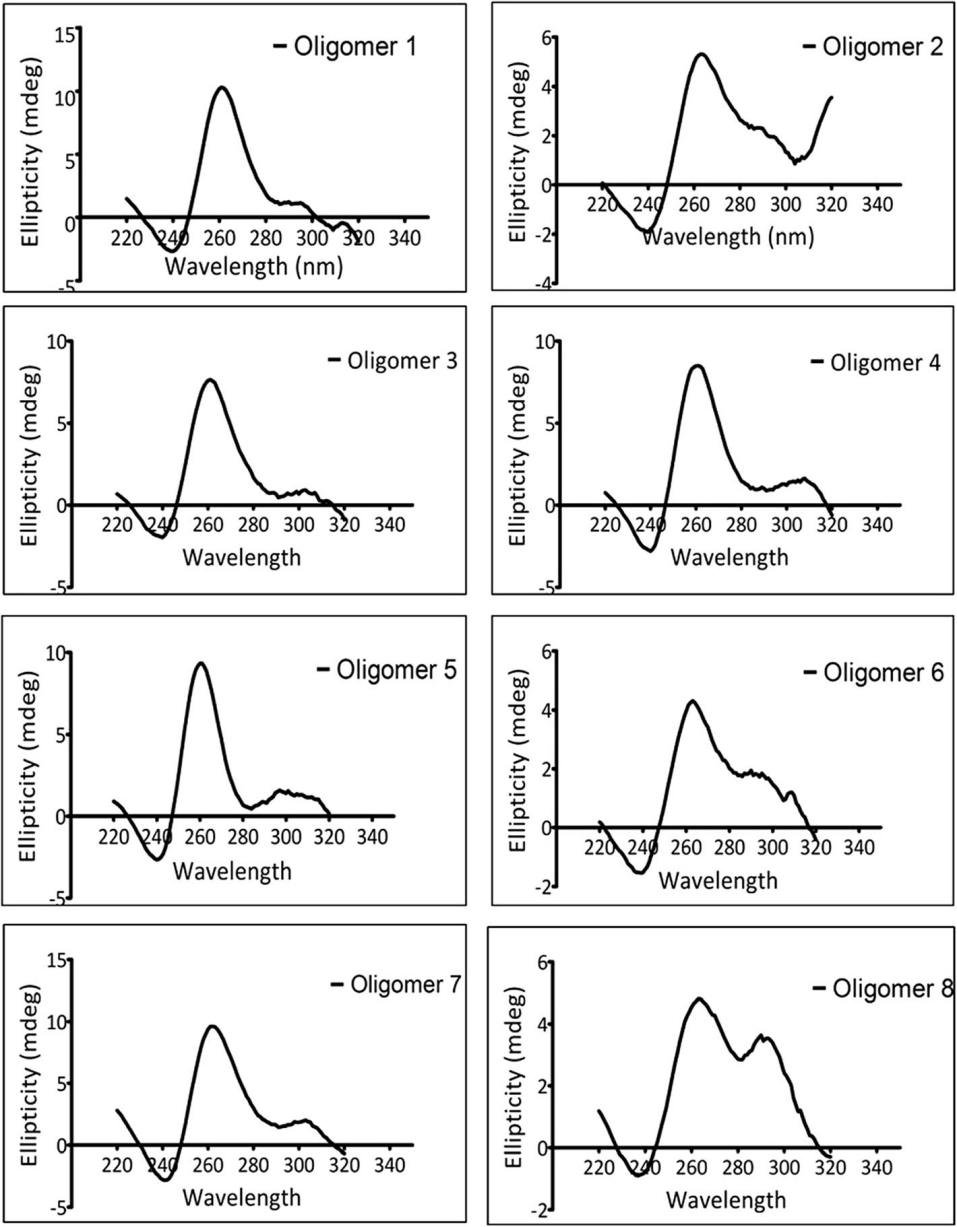
CD spectroscopic analysis of G4-motifs in flanking regions of breakpoints. The spectral signatures of the 8 oligomers chosen from the flanking sites of recombination breakpoints indicate the formation of G4 structures. Oligomers 1,3,4,5, and 7 form G4 s with parallel topology, while oligomers 2,6,8 form G4 s with hybrid topology. (The sequence of the oligomers 1–8 are provided in Additional file 1: Table S2)

CD spectroscopy allows identification of the orientation of the strands in a G4. Parallel G4 s have a positive peak at 260 nm and a negative peak at 240 nm. Antiparallel G4 s have a positive peak at 290 nm and a negative peak at 260 nm. Hybrid G4 s have two positive peaks, one each at 260 nm and 290 nm, and a negative peak at 240 nm. The CD profile of the 8 G4-motifs used in this study indicated the formation of parallel and hybrid G4 structures (Fig. 3 and Additional file 1: Figure S2).

Parallel orientation of strands appears to be a feature common to the G4-motifs associated with recombination. For example, G4-motifs near (a) sites of chromosomal rearrangement in cancer-related genes like HOX11 [33], BCL-2 [34], TCF-3 [35] (b) loci of antigenic variation in *Neisseria gonorrhoeae* [10] and *Treponema pallidum* [36] and (c) the central polypurine tract (cPPT), a dimerization site in HIV-1 [15], formed parallel G4 s in vitro. The ability of such structures to promote strand exchange in vitro was also demonstrated in HIV-1. Moreover, Mre11p, a part of the eukaryotic MRN/X complex involved in repair of DSBs and in meiotic recombination, was reported to have a higher binding affinity for parallel G4 s [13]. As already mentioned, MRN/X complex is known to interact with recombinases in HSV-1 and may be a host factor relevant in HSV-1 recombination [31]. Collectively, these reports reiterate that the parallel G4 s identified herein have a potential functional role in HSV-1 recombination.

**Fig. 4:**
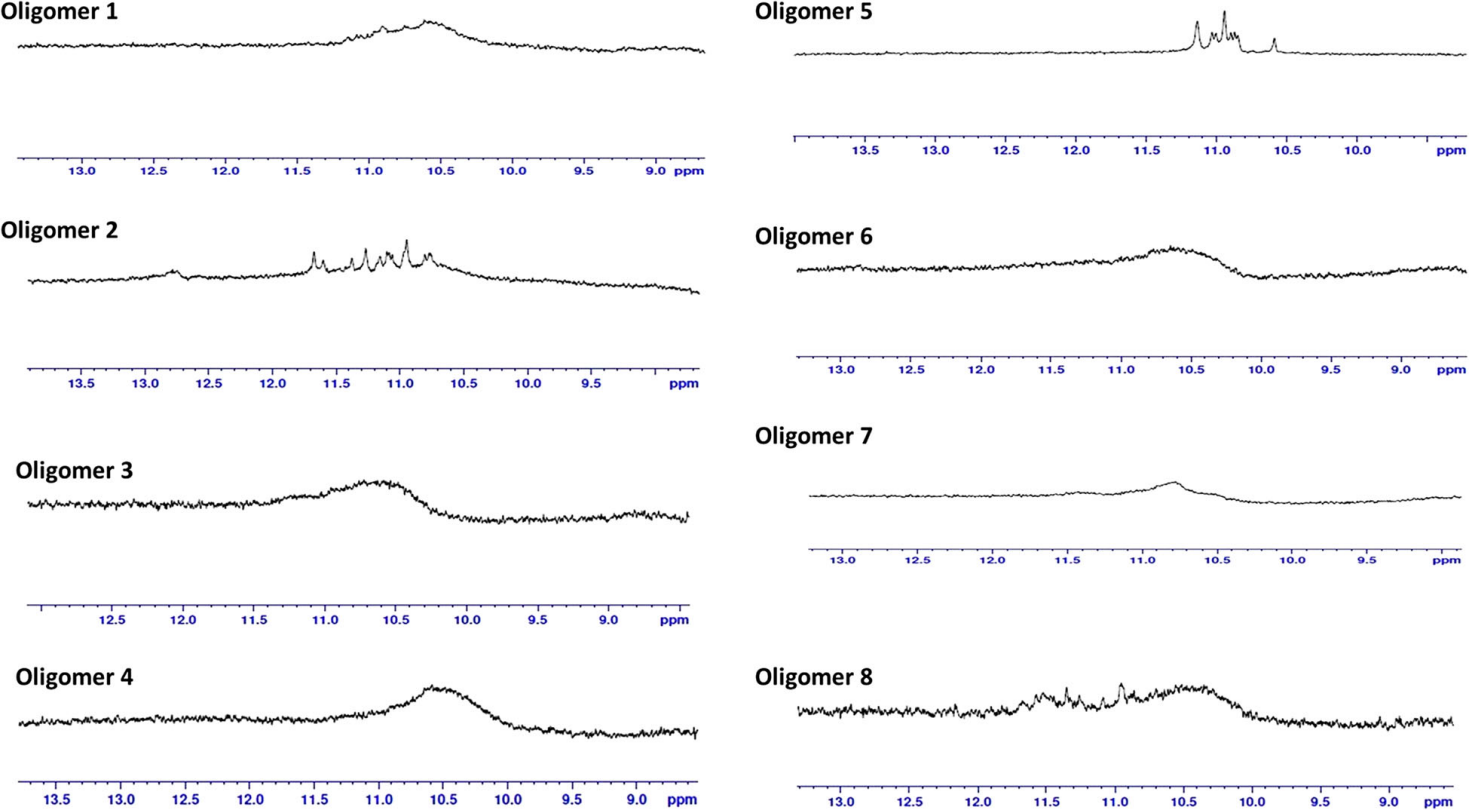
NMR spectroscopic analysis of G4-motifs in flanking regions of breakpoints. The 8 oligomers chosen from the flanking sites of recombination breakpoints form G4 structures in vitro as indicated by the appearance of peaks in the downfield range 10.5–12 ppm in ^1^H NMR. (The sequence of the oligomers 1–8 are provided in Additional file 1: Table S2)

We also confirmed the formation of G4 s by NMR spectroscopy. Peaks appearing in the chemical shift range, 10.5 ppm – 12 ppm, in 1D ^1^H NMR are assigned for the imino protons characteristic of the Hoogsteen base-pairing present in G4 s [37] . All the 8 motifs analyzed formed G4 structures in vitro (Fig. 4 and Additional file 1: Figure S3)*.*

### Recombination breakpoints are located near G4-motifs

**Fig. 5:**
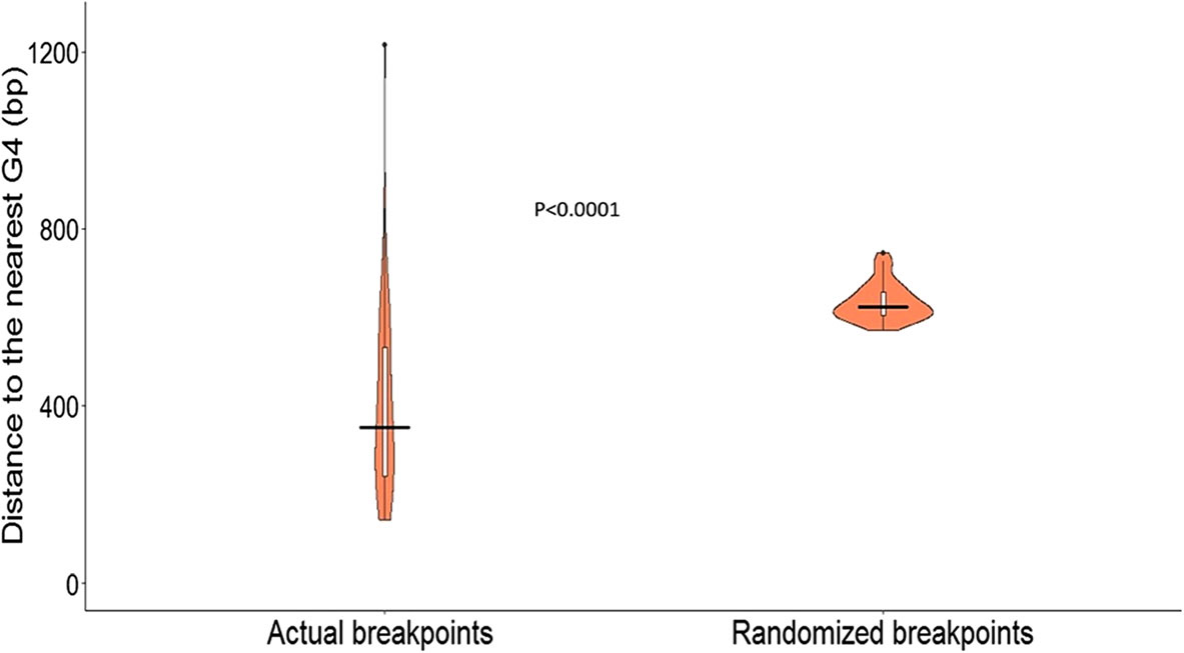
The proximity of recombination breakpoints to G4-motifs is non-random. The median distance of actual recombination breakpoints to their nearest G4-motifs in the genome of the recombinants is significantly lesser than that of the randomly selected breakpoint positions, indicating that, the occurrence of recombination breakpoints near G4-motifs is not stochastic

Encouraged by our initial analyses, we next determined the distance (in bp) of each breakpoint to the 5′ end of its nearest G4-motif in the genome of the respective recombinant. The nearest G4 may lie on either of the sides of the breakpoint and in either of the two strands. For comparison, a null dataset of 750 nucleotide positions, chosen at random using Linux, was generated for each of the 40 recombinant strains (i.e. 750 × 40 = 30,000 breakpoints in 40 recombinant strains) and the distances to their nearest G4-motif were determined. The distances were averaged in the actual dataset and null dataset strain-wise and are plotted in Fig. 5. The median distance of a G4-motif from an actual breakpoint is 351 bps as compared to 625 bps for randomized breakpoints (Fig. 5; *P* < 0.0001), implying that actual (i.e experimentally validated true breakpoints) breakpoints lie closer to G4-motifs than randomly selected points. This finding further corroborates that the proximity of G4-motifs to breakpoints in HSV-1 genome is not by chance.

### G4-motifs themselves are potential hotspots for recombination

**Fig. 6:**
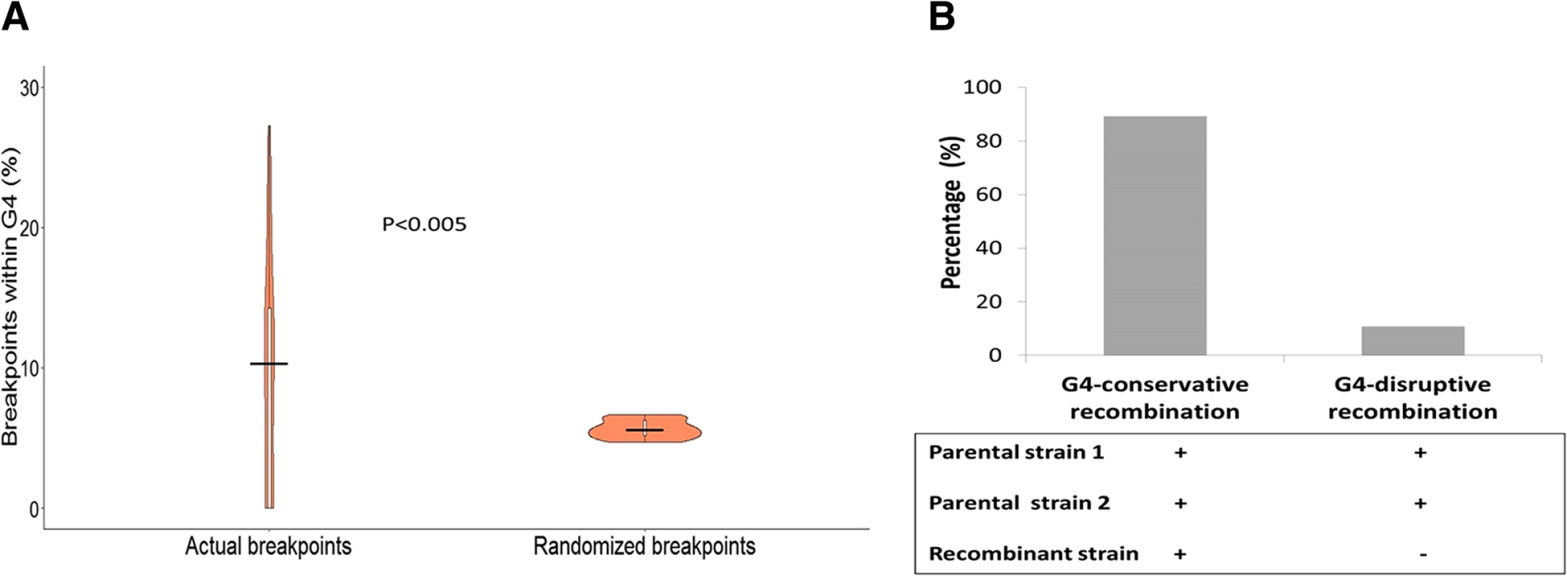
G-quadruplexes are sites of recombination in HSV-1. **a** The proportion of actual breakpoints and randomly selected breakpoints contained within G4-motifs was determined strain-wise and is consolidated in the violin plot. The median proportion of actual breakpoints within G4 motifs was significantly higher than that of randomized breakpoints within G4 motifs, suggesting that G4 motifs in the HSV-1 genome are enriched for breakpoints. **b** When the recombination breakpoint occurs within G4-motif in the parental strains, the G4-motif is often conserved (and not lost) in the process of recombination (i.e. the recombinant strain has a G4-motif in the same region). ‘+’ indicates presence of G4-motif at the breakpoint ‘-‘indicates absence of G4-motif at the breakpoint

Having shown that proximity to G4-motifs is a potential determinant of recombination breakpoints (Fig. 5), we analyzed if the G4-motifs themselves are hotspots for recombination (in other words, we analyzed if breakpoints in HSV-1 are enriched within G4-motifs). The proportion of breakpoints that lie within a G4-motif was determined for the actual dataset (i. e the set of real breakpoints in the 40 strains) strain-wise. Interestingly, we found that about 11% of all breakpoints in the 40 recombinants lie within G4-motifs. Given the high G4 density of the HSV-1 genome (approximately 1/kb), it is possible that breakpoints may fall within G4-motifs by chance. In order to verify this, the proportion was also calculated for the same null datasets (i.e. randomly generated breakpoints or randomized breakpoints; please see methods for details) of the 40 recombinants. The data are summarized in the violin plots shown in Fig. 6a. Interestingly, the proportion of actual breakpoints that lie within a G4-motif was higher as compared to that in randomly generated breakpoints (Fig. 6a; *P* < 0.005). This finding suggests that (a) G4-motifs in the HSV-1 genome are possible hotspots for recombination and (b) the enrichment of breakpoints within G4-motifs is independent of the high G4 density in HSV-1 genomes.

### Recombination occurring within G4-motifs is G4-non-disruptive

Genomic segments encompassing the breakpoints are prone to indels and SNPs; their nucleotide sequence is hence dynamic [38, 39]. Thus, incidence of breakpoints within G4-motifs may affect the integrity of the G4-motif. In this regard, the preferential location of breakpoints within G4-motifs lends us to suppose that disruption of G4-motif may be a fitness cost associated with HSV-1 recombination. To analyze this possibility, we mapped the 577 breakpoint loci in the two parental strains, OD4 and CJ994.We focused on those recombination events where the breakpoint is contained within a G4-motif in both the parental strains. Events were categorized as ‘G4-conservative’ if the motif is retained in the recombinant progeny and as ‘G4-disruptive’, if the G4-motif is lost in the process of recombination. The proportions of G4-conservative and G4-disruptive events are plotted in Fig. 6b. Contrary to the supposition, recombination is predominantly G4-conservative (Fig. 6b). In other words, even when breakpoints are located within G4-motifs, majority of the G4-motifs are conserved (i.e. not disrupted) during recombination. This preservation of G4-motifs in the course of recombination-mediated evolution is interesting and is suggestive of a biological role of G4 s in HSV-1.

### G4-clusters in HSV-1 are hotspots for recombination

**Fig. 7:**
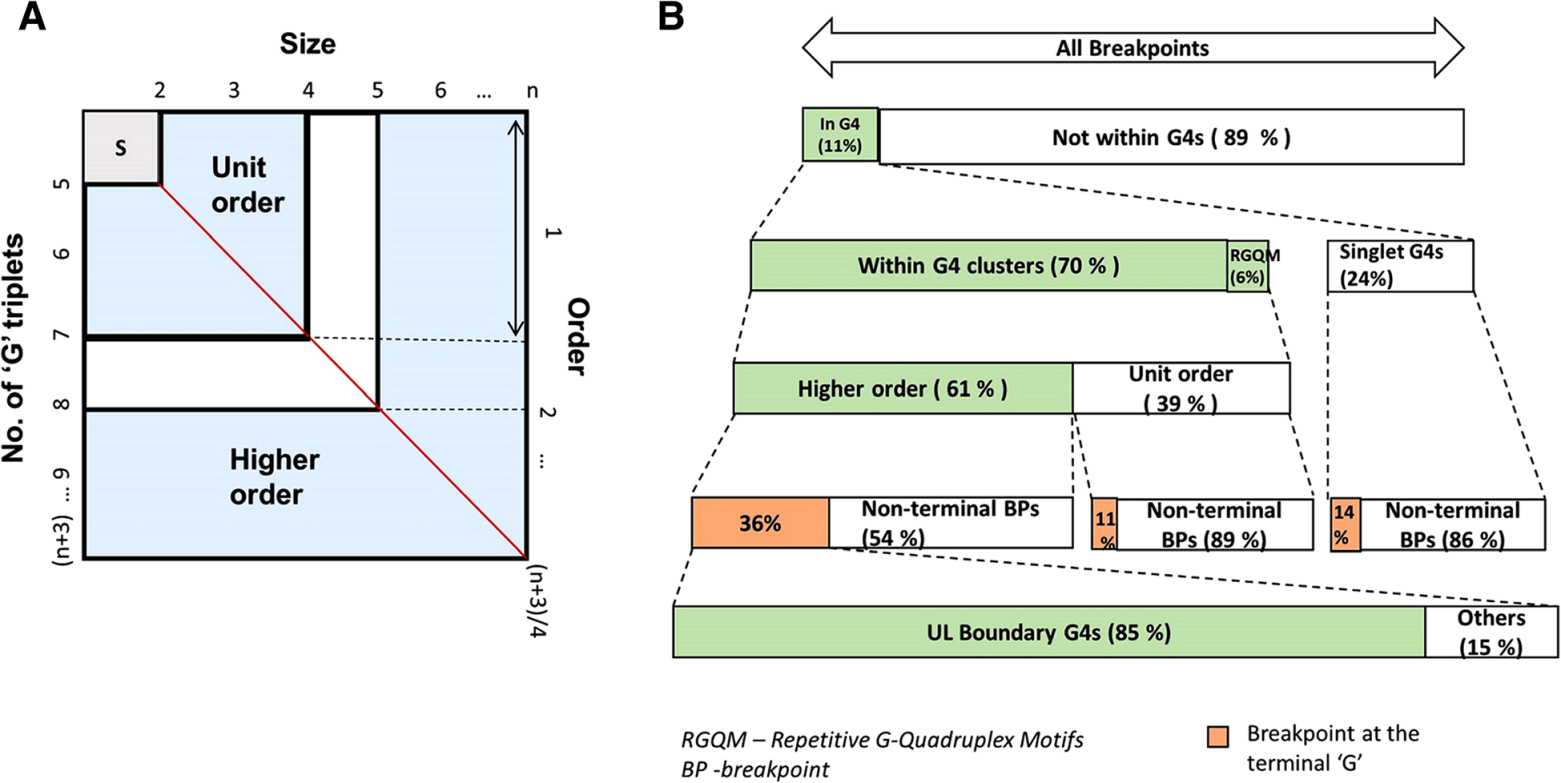
Understanding G4-clusters and unique features of breakpoints within G4-clusters. **a** Understanding G4-clusters: The complete square denotes all G4-motifs. The axes apply only to the blue portions of the square and take only integer values. The subset of all singlet G4-motifs (denoted by ‘S’), characterized by exactly 4 ‘G’ triplets (and hence only 1 independent G4), is represented by the grey portion in the top-left region. The blue portions represent G4-clusters. The red line is a bivariate characteristics curve of G4-clusters, the two variables being, number of G triplets and size. Given the number of ‘G’ triplets, the size of G4 cluster can be determined by extrapolation and vice versa. The white ‘˩’ shaped region merely demarcates the unit order and higher order G4 clusters. **b** Summary of the unique features of breakpoints contained within G4-motifs depicted as a multilevel nested cartoon: The colored bars indicate a unique aspect among the breakpoints present in G4-motifs. Bars are zoomed in at the subsequent level to indicate the features nested within them. The proportion of breakpoints containing the indicated feature is mentioned in each bar

Before explaining our further analyses, it is important here to understand the terms‘G4-cluster’, ‘size of a G4-cluster’ and ‘order of a G4-cluster’ (Fig. 7a). G4-clusters are nucleotide sequences with more than one permutation of G4-motif. They are patches on a polynucleotide where the probability of G4 formation is elevated by the presence of overlapping G4-motifs. For example, five G triplets (unlike the conventional 4 triplets) would give rise to 2 overlapping G4-motifs, when counted conservatively without skipping triplets, and would constitute a G4-cluster. The size of a G4-cluster is the total number of overlapping G4-motifs it contains and takes a minimum value of 2. As can be noted from the above example, the size is a function of the number of G triplets in the G4-cluster (Fig. 7a). The order of a G4-cluster is the number of non-overlapping G4-motifs it contains. Two subclasses can be recognized on the basis of order of G4-clusters: Unit-order and higher-order G4-clusters. Unit-order G4-clusters have an order of 1. In other words, although multiple overlapping permutations of G4-motifs may exist, only one G4 structure can truly form at any given point of time in a unit order G4-cluster. In higher-order G4-clusters, multiple G4 structures form simultaneously in tandem, giving a “beads-on –a string” appearance [40]. Such higher-order G4 structures have been described for human telomeric G4-motifs [41]. The characteristics curve of G4-clusters shown in Fig. 7a summarizes the inter-relationship between its G triplets, size and order.

We analyzed the subset of breakpoints lying within G4-motifs (i.e. a total of 64 breakpoints) and identified some unique features (Fig. 7b). Firstly, majority (about 76%) of these breakpoints are harbored within G4-clusters (Fig. 7a) as compared to in individual G4-motifs (24%). Secondly, some of the breakpoint-containing G4-clusters were the repetitive G-quadruplex motifs (RGQMs), characterized earlier in HHVs [19]. RGQMs are G4-forming repetitive sequences with iterations across the genome; their functional roles are however unknown. Our finding, suggests a potential role for RGQMs in virus recombination. Thirdly, among the breakpoints present within G4-clusters, most of them (about 61%) are borne in higher-order G4-clusters (Fig. 7b). Recombination in HSV-1 is closely intertwined with replication. G4 s in HSV-1 have been shown to stall the progression of DNA polymerase under in vitro conditions [21]. We hypothesize the higher-order G4 s to potentially exacerbate the polymerase stalling, leading to nicking and onward to introduction of double strand breaks and recombination.

### The terminal guanosine of G4-cluster at the boundary of the U_L_ region is the commonest breakpoint

**Fig. 8:**
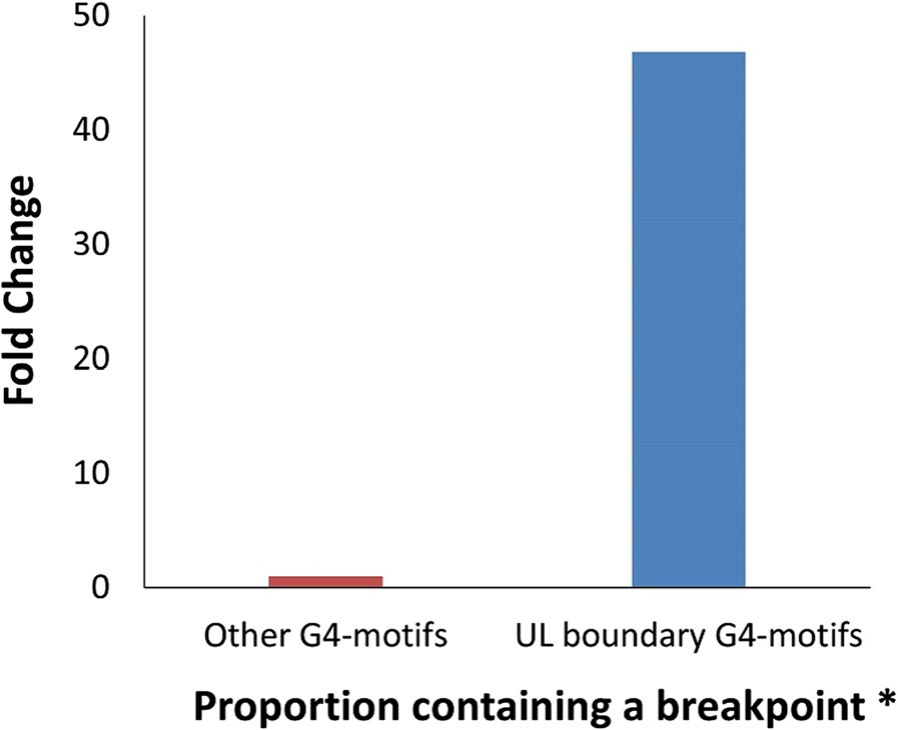
G4-motifs at the boundary of the U_L_ segment are hotspots of recombination. G4-motifs at the boundary of U_L_ are approximately 46-fold more enriched for recombination breakpoints as compared to other G4-motifs, making them the most common G4-motifs to contain a breakpoint in HSV-1 recombination. *The proportion of G4-motifs containing a breakpoint is significantly higher among the UL boundary G4-motifs than among other G4-motifs (*P* < 0.0001; Chi-squared test)

We observed the two boundary nucleotide positions of U_L_ segment to recur as breakpoints (Fig. 7b). Fascinatingly, these two common breakpoint loci are also the terminal guanosines of two higher-order G4-clusters, present one on each boundary of U_L_ (henceforth known as U_L_ boundary G4-clusters). Importantly, recombination breakpoints are approximately 45-fold enriched in the U_L_ boundary G4-cluster as compared to other G4-motifs in the HSV-1 genome (Fig. 8); this finding indicates that the G4-cluster at the U_L_ boundary may contribute significantly to recombination in HSV-1.

A possible explanation for the overrepresentation of the two U_L_ boundary G4-clusters in recombination may lie in their genomic position with respect to HSV-1 oriL (the origin or replication that lies in the U_L_ segment). The two U_L_ boundary G4-clusters are present on both sides of oriL. We speculate that the higher-ordered nature (their order value is 9) of the two U_L_ boundary G4-clusters may represent a formidable challenge for the viral polymerase that stalls at the very first nucleotide (i.e. the terminal “G” nucleotide of both the U_L_ boundary G4-clusters), thus making the oriL-proximal terminus of these G4-clusters a common recombination locus.

The junctions of the unique and repeat segments of the HSV-1 genome are known to be recombinogenic [42, 43]. While they are known to be preferred sites of intramolecular recombination leading to duplication and inversion of genomic segments, no such precedence of these sites in intermolecular recombination is known. Our report identifies the terminal “G” nucleotides which are part of the two U_L_ boundary G4-clusters at the junction of the unique and repeat segments of HSV-1 to be common sites of intermolecular recombination.

In sum, our computational analyses strongly argue in favour of an association between G4 s and recombination breakpoints in the HSV-1 genome. However, we have not attempted to identify the possible underlying mechanisms. It is possible that some breakpoints could have been missed due to drawbacks in currently available sequencing techniques; although this number may be small, it represents another limitation of this study.

## Conclusion

Association between G4 s and recombination has not been previously reported among DNA viruses. Here, we report multiple lines of evidences linking G4-motifs and recombination in HSV-1 genomes. We identified that recombination landscape is closely associated with the density of G4-motifs in the HSV-1 genome (Fig. 1). Encouraged by the spatial association between G4-motifs and recombination, we zeroed in on the individual breakpoints in the 40 recombinant strains and analyzed them on the basis of two fundamental questions (a) how do the genomic segments containing recombination breakpoints differ from the rest of the genome in terms of G4 demography? (b) How close are recombination breakpoints to G4-motifs? These questions allowed us to address two distinct aspects of the relationship between recombination breakpoints and G4-motifs, making our approach two-pronged. The former is based on a spatial association between G4-motifs and recombination breakpoints, while the latter is based on a one-dimensional variable of length. Our analysis revealed a selective enrichment of G4-motifs in 500 bp regions flanking the recombination breakpoints in HSV-1 (Fig. 2). Oligonucleotides from a subset of predicted G4-motifs in the flanking sites of breakpoints formed secondary structures in vitro (Figs. 3 and 4). We noted that recombination breakpoints of HSV-1 are specifically located close to G4-motifs (roughly 350 bp) as compared to randomly selected points on the HSV-1 genome (Fig. 5). An intriguing answer to question (b) is breakpoints can lie as close as within G4-motifs themselves. Interestingly, such type of recombination events (i.e. breakpoints contained within G4-motifs) have a more than expected representation in the recombination scenario of the 40 strains analyzed in our study (Fig. 6a). This finding emphasizes a role for G4 s in recombination in HSV-1. In addition, we found that G4-clusters are hotspots for recombination in HSV-1. Furthermore, we noted that breakpoints often lie in the terminal nucleotide positions of higher-order G4-clusters (Fig. 7). Importantly, the two most common recombination breakpoints of HSV-1, the boundary nucleotides of U_L_ segment, are the terminal nucleotides of higher-order G4-clusters, indicating a significant role for higher-order G4-clusters in HSV-1 recombination (Fig. 8). Such roles for higher-order G4 s in microbial genomes have not been reported thus far. Our work provides a novel view of HSV-1 evolution which may be important in understanding its epidemiology, replication and virulence characteristics. Our findings also shed light on hitherto unknown roles for G4 s in the genomes of DNA viruses.

### Methods

#### Retrieval of sequences

The whole genome sequences of strain 17, parental (OD4 and CJ994) and the 40 recombinant strains were retrieved from NCBI in FASTA format using the accession numbers reported by Lee et al [23] (Additional file 1: Table S1).

#### Identification of G4-motifs and computation of G4 density

The genomes of the 40 recombinant strains were mined for G4-motifs conforming to the motif, G_3_N_1-7_G_3_N_1-7_G_3_N_1-7_G_3_,using Quadparser [24]. Both strands of the genome were searched for G4-motifs. The program lists the nucleotide positions and the sequences of the identified G4-motifs in the output. This output was used for identification of (a) the G4-motif nearest to a given breakpoint in a strain (b) the G4-motifs harboring recombination breakpoints in a strain. Only non-overlapping G4-motifs were considered in our analysis.

G4 density is defined as the total number of non-overlapping G4-motifs present per kilobase of the sequence analyzed. In other words, it is the total number of non-overlapping G4-motifs normalized to the length of the input sequence. It is computed strand-wise and then averaged for both the strands. The G4 density was calculated for the following. (a) 100 bp windows of the genome of strain 17, the reference strain (b) flanking regions of breakpoints in the genome of recombinants and the rest of their genome (i.e. the entire genome other than the flanking regions) (c) randomized sequences of the flanking regions.

- Sliding window analysis: A nucleotide window of 100 bp, advancing by one basepair, was slid along the length of the genome of strain 17 using an in-house program [19]. A total of 152,162 100 bp windows were generated. The window sequences were then input to Quadparser and their G4 densities were computed.
- Analysis of 500 bp region flanking the breakpoints in the recombinants’ genome: The whole genome sequences of each of the 40 recombinants was input to Range Extractor tool of Sequence Manipulation Suite, an online sequence analysis platform, for extraction of the 500 bp region flanking each of their respective breakpoints on either sides. If the flanking sequences of two consecutive breakpoints overlap, a single segment starting from (predecessor breakpoint - 500) to (successor breakpoint + 500) was considered to avoid double-counting of G4-motifs and the length of the region of overlap. The number of G4-motifs in the flanking regions of all breakpoints in a strain was summed up strand-wise, normalized to the total length of flanking regions and averaged to compute the G4 density of the flanking regions. Likewise, the segments of the rest of the genome (i.e. other than the flanking regions of the breakpoints) were also extracted from the respective recombinant’s genome and the G4 density was calculated.
- Randomization of the 500 bp flanking regions: The 500 bp flank sequences of all breakpoints in the 40 strains were randomized 5 times without altering the overall nucleotide composition using Bioedit [44] with the software’s default randomization parameter of 10,000 shuffles. The G4 density of the randomized sequences was calculated for each randomization trial as described earlier and averaged over 5 trials strain wise.

#### Generation of random breakpoints

If recombination were a chance event, the breakpoints would be uniformly distributed throughout the genome and be independent of G4-motifs. To test this null hypothesis, random breakpoints were generated for each recombinant genome. The syntax used for generating random numbers within a defined range in Linux is as follows: **shuf –i** < *lower limit –upper limit* > **−n** < *number of random numbers* > **−o** < *output file name* >. The output is a *.txt* file. Seven hundred and fifty random breakpoints were generated for each of the 40 recombinant strains. These comprise the set of ‘randomized breakpoints’ referred to in Figs. 5 and 6.

#### Data analysis, graphical representation and statistics

Microsoft Excel was used for analysis of data and plotting of bar graphs. Violin plots were generated using the software R. The R packages used were ggplot, forcats, and R ColorBrewer. CD spectral curves were plotted using Graphpad Prism 5.0. Origin 9.1 was used for plotting of Fig. 1. Figure 7 was created using MS PowerPoint. Unless mentioned otherwise, statistical significance was determined using Wilcoxson matched-pairs signed rank test in Graphpad Prism 5.0. *P* values less than 0.05 were considered significant.

#### Circular dichroism spectroscopy

Among the G4-motifs present in the 500 bp flanking region of breakpoints of all 40 strains, 8 were selected at random using MS Excel. The sequences of the 8 G4-motifs chosen are listed in Additional file 1: Table S2. The oligonucleotides were purchased from Integrated DNA Technologies (IDT) for in vitro analyses.

Oligonucleotides prepared at 10 μM concentration in a buffer containing sodium cacodylate (10 mM) and KCl (100 mM) were heated at 95 °C for 5 min and cooled to room temperature on standing. A sample containing only the buffer components and treated in the same manner was used as blank. CD spectroscopy was performed using J 815 spectrophotometer (JascoInc, Japan) and a quartz cuvette with a pathlength of 1 mm. The following parameters were used for obtaining the spectra (a) Temperature: 20 °C (b) Wavelength range: 220 nm–320 nm (c) Accumulations: 3 (d) Bandwidth: 0.5 nm (e) Step size: 1 nm (f) Time per point: 1 s.

#### NMR spectroscopy

Oligonucleotides (Additional file 1: Table S2) at a concentration of 300 μM were prepared in 20 mM phosphate buffer (pH 7.0) containing 100 mM KCl and 10% D_2_O (v/v), heated to 95 °C and allowed to cool slowly to room temperature before measurement of spectra. 1D ^1^H NMR spectra were recorded at 20 °C on Bruker Avance III spectrometer operating at 500 MHz field strength. Topspin 3.5 was used for data acquisition, data processing and plotting of spectra.

## Additional file

Additional file 1: **Table S1.**List of accession numbers of HSV-1 strains analyzed in this study. **Table S2.** List of G4-motifs chosen from the flanking sites of recombination breakpoints for biophysical characterization. **Table S3.** Details of the G4-motifs chosen for biophysical characterization. **Figure S1.** Enrichment of G4-motifs in flanking sites of recombination breakpoints is non-random. **Figure S2.** CD spectroscopy of negative control oligonucleotide. **Figure S3.** NMR spectroscopy of negative control oligonucleotide. (DOCX 501 kb)

## Abbreviations

G4: G-Quadruplex
HHV-1: Human Herpes Virus-I
HSV-1: Herpes Simplex Virus-I
RGQM: Repetitive G-Quadruplex Motif
