# Supplementary material for: G-quadruplexes may determine the landscape of recombination in HSV-1
Source: BMC Genomics. 2019 May 16;20:382. doi: 10.1186/s12864-019-5731-0 (PMC6524338; doi:10.1186/s12864-019-5731-0)
Supplement: Supplementary file 1 — Table S1.List of accession numbers of HSV-1 strains analyzed in this study. Table S2. List of G4-motifs chosen from the flanking sites of recombination breakpoints for biophysical characterization. Table S3. Details of the G4-motifs chosen for biophysical characterization. Figure S1. Enrichment of G4-motifs in flanking sites of recombination breakpoints is non-random. Figure S2. CD spectroscopy of negative control oligonucleotide. Figure S3. NMR spectroscopy of negative control oligonucleotide. (DOCX 501 kb) [file 12864_2019_5731_MOESM1_ESM.docx]

**SUPPORTING INFORMATION**

**G-QUADRUPLEXES MAY DETERMINE THE LANDSCAPE OF RECOMBINATION IN HSV-1**

Nandhini Saranathan^a^, Banhi Biswas^a^, Anupam Patra^b^, Perumal Vivekanandan^a*^

^a^Kusuma School of Biological Sciences, Indian Institute of Technology Delhi, New Delhi, India

^b^International Centre for Genetic Engineering and Biotechnology, New Delhi, India

^*^Corresponding author. Email: [vperumal@bioschool.iitd.ac.in](mailto:vperumal@bioschool.iitd.ac.in) Tel : 91-11-26597532 ;

Fax: 91-11-26582037

**Table S1. List of accession numbers of HSV-1 strains analyzed in this study**

| **Designation in this study** | **Name of the strain** | **Accession Number** |
| --- | --- | --- |
| Reference strain | 17 | NC_001806 |
| Parental strains | OD4 | JN420342 |
|  | CJ994 | KR011283 |
| Recombinant strains | 3M | KR011282 |
|  | 4M | KR011278 |
|  | 8S | KR011280 |
|  | 11M | KR011294 |
|  | 12-12-2 | KR011298 |
|  | 12-12-67 | KR011286 |
|  | 16S | KR011303 |
|  | 19Lsyn | KR011293 |
|  | 20L | KR011289 |
|  | 26S | KR011308 |
|  | 27S | KR011297 |
|  | 31XL | KR011304 |
|  | 34L | KR011275 |
|  | 36L | KR011279 |
|  | 47M | KR011305 |
|  | 57M | KR011276 |
|  | 65M | KR011312 |
|  | 66S | KR011281 |
|  | 76S | KR011300 |
|  | 78S | KR052507 |
|  | 81L | KR052508 |
|  | 82S | KR011307 |
|  | 83M | KR011310 |
|  | 5-4-2 | KR011311 |
|  | 5-5-2 | KR011295 |
|  | 10-2-3 | KR011274 |
|  | 10-6-1 | KR011296 |
|  | 10-6-3 | KR011284 |
|  | 2-5-3 | KR011292 |
|  | 10-5-1 | KR011301 |
|  | 10-6-2 | KR011306 |
|  | 10-11-2 | KR011287 |
|  | 10-1-2 | KR011302 |
|  | 5-4-2 | KR011311 |
|  | 10-2-2 | KR011277 |
|  | 2-4-2 | KR011288 |
|  | 10-7-1 | KR011290 |
|  | 10-11-3 | KR011309 |
|  | 5-1-1 | KR011299 |
|  | 10-14-1 | KR011291 |

**Table S2. List of oligonucleotides chosen for biophysical characterization.**

| **Name** | **Sequence (5’ to 3’)** | **Length (nt)** |
| --- | --- | --- |
| Oligomer 1 | GGGTTGGGTGGGGGAGTGTGGG | 22 |
| Oligomer 2 | GGGATCGTGCGGGCCGGGGGTCGCCGGGG | 29 |
| Oligomer 3 | GGGGCGGGAGGGGGCGAGGG | 20 |
| Oligomer 4 | GGGTGGTGGGCGAGGGGGGAGGGGG | 25 |
| Oligomer 5 | GGGCCGGGTGGGCGGGG | 17 |
| Oligomer 6 | GGGGGGGGGGAGCGCGGGCCGGG | 23 |
| Oligomer 7 | GGGCTGAGGGGAAAAAAGGGGGGGCGGG | 28 |
| Oligomer 8 | GGGGGGGTTGGGGTGGGGGTTGGGG | 25 |
| Negative control (CD) | GCTATGGCTTGCTATGGCTTGCTAT | 25 |
| Negative control (NMR) | GAGAGAGATAGAGAGAGA | 18 |

**Table S3: Details of the G4-motifs chosen for biophysical characterization**

| **Oligomer Name** | **Strain name** | **Strand ^a^** | **Frequency** | **Position ^b^** | **Is it present in strain 17? Yes/No** |
| --- | --- | --- | --- | --- | --- |
| Oligomer 1 | 5-5-2 | Primary | 1 | 117346 | No |
| Oligomer 2 | 10-11-2 | Primary | 1 | 7932 | Yes |
|  |  | Complementary | 1 | 118107 |  |
| Oligomer 3 | 5-5-2 | Primary | 8 | 150958,150975, 150992,151009, 151026,151247, 151264,151196 | Yes |
|  |  | Complementary | 6 | 126644,126661, 126899,126916, 126933,126950 |  |
| Oligomer 4 | 65M | Primary | 1 | 50515 | Yes |
| Oligomer 5 | 81L | Complementary | 1 | 117655 | Yes |
| Oligomer 6 | 5-1-1 | Primary | 1 | 8664 | Yes ^c^ |
|  |  | Complementary | 1 | 117740 |  |
| Oligomer 7 | 10-11-2 | Primary | 1 | 123139 | Yes ^c^ |
|  |  | Complementary | 1 | 2905 |  |
| Oligomer 8 | 5-5-2 | Primary | 1 | 4000 | Yes ^c^ |
|  |  | Complementary | 1 | 122080 |  |

^a^ Primary strand refers to the sequence submitted in NCBI.

^b^ The nucleotide position of the start of the G4-motif in the 5’ -3’ orientation is indicated. The positions indicated are with respect to the recombinant genome.

^c^ Except for 1 SNP in the loop, the G4-motif aligns with the genome of strain 17 (This means that these G4- motifs are still intact in strain 17)

**Figure S1. Enrichment of G4 motifs in flanking sites of recombination breakpoints is non-random.**

**A**


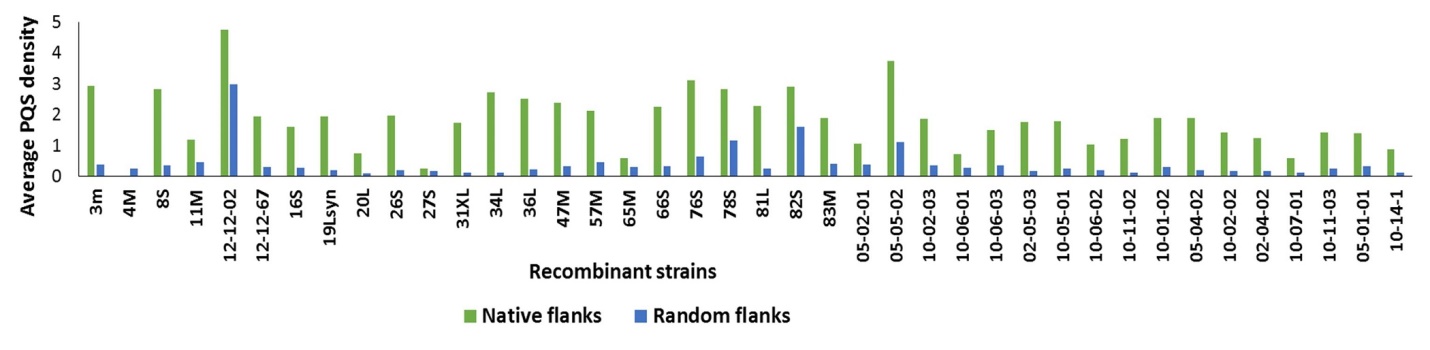

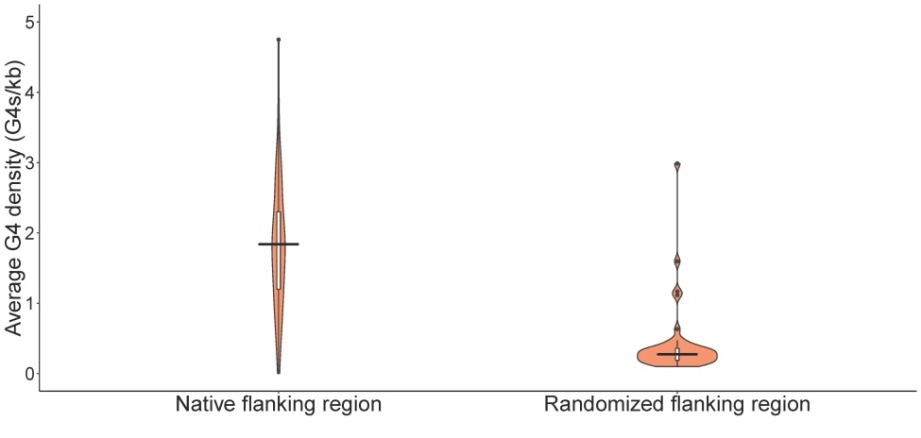


**B**

P<0.0001

**LEGEND**

To ascertain whether the 1-kb bin centered on recombination breakpoints are enriched for G4 motifs independent of their GC-content, the sequences were scrambled 5 times without changing the overall nucleotide composition.

(A) Strain-wise distribution of G4 densities in the native and randomized flanking regions of breakpoints: Most native sequences have a higher average G4 density as compared to randomized sequences, indicating that the presence of G4 motifs in flanking regions is not merely because of the differences (if any) in the nucleotide composition of the regions flanking the breakpoint. (B) The violin plot summarizes the data from all 40 strains.

**Figure S2. CD spectral signature of negative control oligonucleotide.**


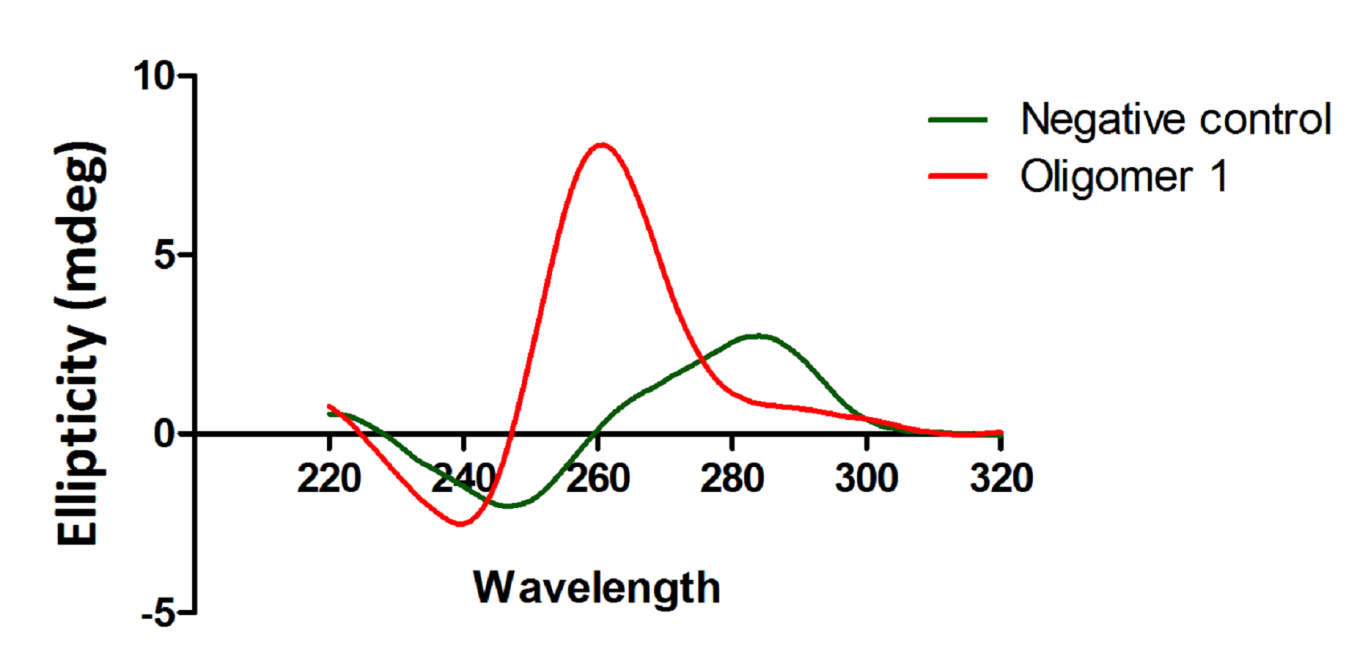


**LEGEND**

A 25-mer with a random nucleotide sequence that doesn’t conform to a G4-motif was used as a negative control in CD spectroscopy. As expected, it did not show peaks characteristic of G-quadruplexes. Oligomer 1 was used as a positive control in this experiment.

**Figure S3. NMR spectroscopy of negative control oligonucleotide**

**A.**


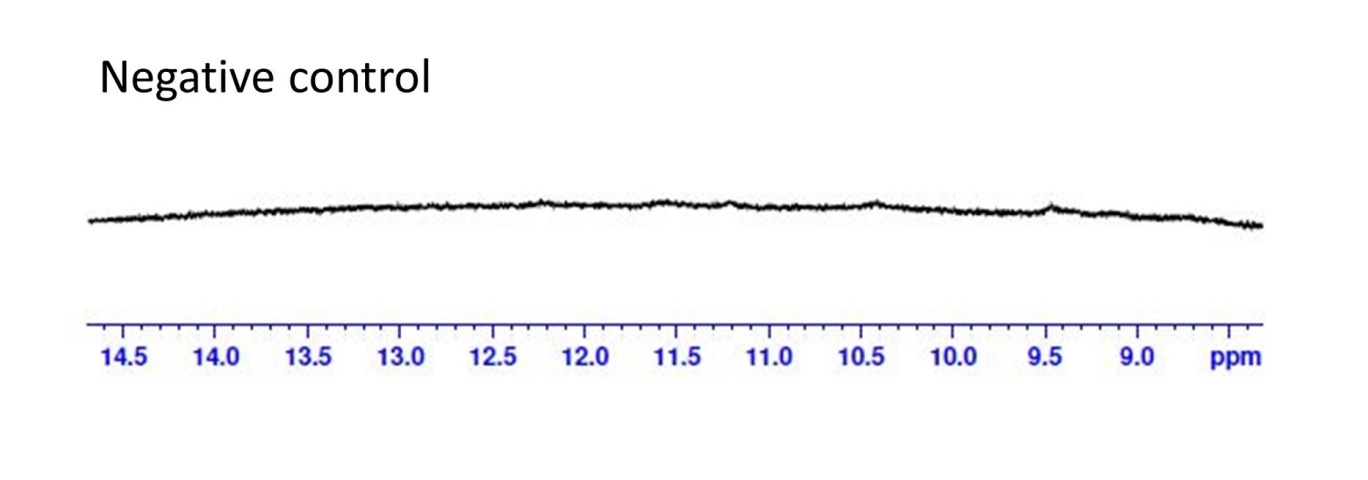


**B**

**
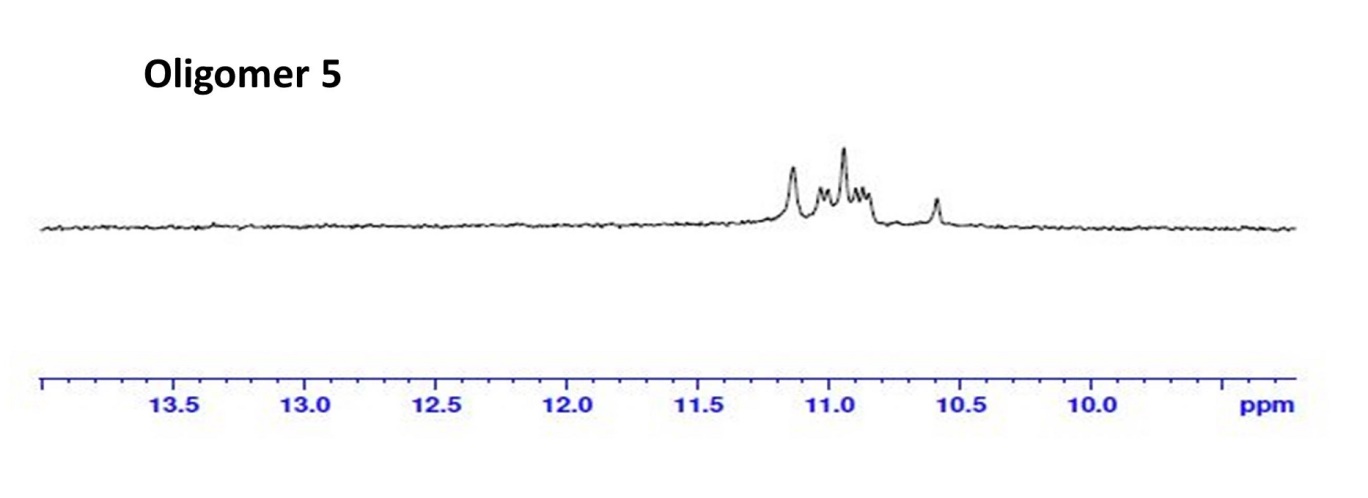
**

**LEGEND**

An 18-mer oligonucleotide that doesn’t conform to a G4-motif was used as a negative control for NMR spectroscopy. Peaks between 10.5 and 12 ppm, characteristic of Hoogsteen bonds, were absent. Oligomer 5 was used as a positive control.
